# Hippocampal gray matter increases following multimodal psychological treatment for combat‐related post‐traumatic stress disorder

**DOI:** 10.1002/brb3.956

**Published:** 2018-04-06

**Authors:** Oisin Butler, Gerd Willmund, Tobias Gleich, Jürgen Gallinat, Simone Kühn, Peter Zimmermann

**Affiliations:** ^1^ Max Planck Institute for Human Development Center for Lifespan Psychology Berlin Germany; ^2^ Center for Military Mental Health Military Hospital Berlin Berlin Germany; ^3^ Clinic for Psychiatry and Psychotherapy Campus Charité Mitte Charité University Medicine Berlin Germany; ^4^ Clinic and Policlinic for Psychiatry and Psychotherapy University Clinic Hamburg‐Eppendorf Hamburg Germany

**Keywords:** adult neurogenesis, post‐traumatic stress disorder, psychiatry, stress

## Abstract

**Introduction:**

Smaller hippocampal volumes are one of the most consistent findings in neuroimaging studies of post‐traumatic stress disorder (PTSD). However, very few prospective studies have assessed changes in hippocampal gray matter prior to and following therapy for PTSD, and no neuroimaging studies to date have longitudinally assessed military populations.

**Methods:**

A pilot study was conducted, assessing patients with combat‐related PTSD with structural MRI. Participants were then assigned either to a treatment group or waiting‐list control group. After the treatment group received multimodal psychological therapy for approximately 6 weeks, both groups completed a second neuroimaging assessment.

**Results:**

Region‐of‐interest analysis was used to measure gray matter volume in the hippocampus and amygdala. There was a group by time interaction; the therapy group (*n* = 6) showed a significant increase in hippocampal volume and a nonsignificant trend toward an increase in amygdala volume following therapy, while no change was observed in the waiting‐list group (*n* = 9).

**Conclusions:**

This study provides initial evidence for increases in gray matter volume in the hippocampus in response to therapy for combat‐related PTSD.

## 1 INTRODUCTION

Post‐traumatic stress disorder (PTSD) is a complex and debilitating psychiatric disorder caused by exposure to traumatic events. Clinically, PTSD is characterized by symptoms of hyperarousal, avoidance, and re‐experiencing, including intrusive memories and visual flashbacks of the traumatic event (Gonzalez & Chiodo, 2015). PTSD is unique among psychiatric disorders as the diagnostic criteria require exposure to an identifiable traumatic event.

Military deployment and combat exposure are specific and highly relevant instantiations of repeated stress and trauma. In recent years, military and political conflicts across the globe have increased, resulting in greater numbers of individuals experiencing combat and continuous exposure to extreme stress. Rates of PTSD in combat‐exposed military populations appear to be consistently higher than in comparable civilian populations (Bandelow et al., 2012; Fear et al., 2010; Kessler et al., 2014; Thomas et al., 2010; Trautmann et al., 2016; Wittchen et al., 2012). In addition, individuals with combat‐related PTSD appear to show higher levels of psychopathology (Frueh, Hamner, Cahill, Gold, & Hamlin, 2000) and higher rates of suicide than civilian populations (Kemp & Bosarte, 2012).

At a neural level, trauma and stress exposure have been associated with alterations in the function and structure of the brain. In animal models, chronic stress exposure has been shown to reduce gray matter volume via reductions in dendritic branching and neurogenesis (Lupien, McEwen, Gunnar, & Heim, 2009; McEwen & Morrison, 2013). The hippocampus is particularly vulnerable to the effects of chronic stress, as it has been shown to be highly sensitive to the effect of glucocorticoids (Sapolsky, Krey, & McEwen, 1985; Sapolsky, Uno, Rebert, & Finch, 1990), the main hormone involved in the chronic stress response.

Neuroimaging research on PTSD has reported smaller regional gray matter volumes, including the hippocampus and amygdala (Karl et al., 2006; Kitayama, Vaccarino, Kutner, Weiss, & Bremner, 2005; Kühn & Gallinat, 2013; Smith, 2005). A key role for these regions in PTSD symptomatology is supported by their well‐established roles in memory (Squire, 1992) and the detection of affective or threatening stimuli (Fitzgerald, Angstadt, Jelsone, Nathan, & Phan, 2006; Öhman, 2005). A neurocircuitry model of PTSD proposes that there is a failure to inhibit a fear reaction in response to threat, in part through reduced bottom‐up control by the hippocampus on the amygdala (Rauch, Shin, & Phelps, 2006; Shin, Rauch, & Pitman, 2006).

Functionally, the hippocampus is a key region in learning and memory (Squire, 1992). Hippocampal volume has been shown to affect contextual fear conditioning in humans (Pohlack et al., 2012), and smaller hippocampal volume has been associated with increased risk (Gilbertson et al., 2002), longer duration (Apfel et al., 2011; Chao, Yaffe, Samuelson, & Neylan, 2014), and poorer treatment response (Rubin et al., 2016; van Rooij et al., 2015) in PTSD. In animal studies, stress‐induced alterations in hippocampal morphology have been linked to increases in anxiety and depression‐like behavior (Lagace et al., 2010), and reductions in cognition and memory (Kim, Diamond, Haven, & Blvd, 2002; McEwen & Sapolsky, 1995). Dysfunctions in memory, specifically intrusive memories or flashbacks, are one of the core symptoms of PTSD, while one of the key objectives of therapeutic interventions for PTSD is to reduce the frequency and vividness of these intrusions.

One may postulate that therapeutic interventions for PTSD will increase hippocampal volume. However, there is a paucity of longitudinal or prospective neuroimaging on PTSD. To our knowledge, only two studies to date have longitudinally assessed PTSD patients with structural MRI prior to and following therapy, and no studies to date have assessed combat‐related PTSD. In a study by Vermetten, Vythilingam, Southwick, Charney and Bremner (2003), long‐term treatment with the antidepressant paroxetine was shown to increase hippocampal volume and improve memory, while in a study by Lindauer et al. (2005), no increase in hippocampal volume was observed following psychotherapy. It remains an open question if and how gray matter changes following psychological therapeutic intervention for combat‐related PTSD.

We conducted a pilot longitudinal study to explore changes in gray matter and psychological symptoms following psychological therapy. We recruited soldiers with combat‐related PTSD and assessed them with psychological questionnaires and clinical interviews and structural magnetic resonance imaging (MRI) at two time points. Following the first assessment, patients were randomly assigned to either a therapy group or a waiting‐list control group. Following completion of therapy, approximately 6 weeks later, both groups of patients were again assessed with psychological questionnaires and structural MRI. Changes in gray matter were assessed using voxel‐based morphometry, and gray matter values from the amygdala and hippocampus were extracted using a region‐of‐interest approach. Regions were selected on a theoretical basis, as alterations in these areas are commonly observed in PTSD populations, and also on a practical basis, as they are discrete and clearly defined anatomical regions.

To our knowledge, this is the first longitudinal study to compare patients with combat‐related PTSD before and after therapy to a waiting‐list control group. We hypothesized that the therapy group would show both improved symptom scores and increased gray matter following therapy compared with the control group.

## 2 MATERIALS AND METHODS

### 2.1 Participants

Twenty soldiers with combat‐related PTSD, prior to onset of therapy, were recruited from the German Armed Forces. All participants were male and had been deployed overseas to areas of conflict. As inclusion criteria, participants were screened by a multidisciplinary team of clinicians, including clinical psychologists and psychiatrists, for the presence of mission‐related trauma within the last 2 years and for a current diagnosis of PTSD according to ICD‐10 criteria. As exclusion criteria, participants were also screened for current or previous comorbid axis I psychiatric disorders (American Psychiatric Association, 2000) using the Mini‐DIPS (Diagnostisches Kurz‐Interview bei psychischen Störungen; Margraf, 1994; Sheehan et al., 1998), use of psychotropic medication and MRI contraindications. The local ethics committee of Charité University Clinic, Berlin, Germany, approved the study, and written informed consent was obtained from each participant prior to participation, in line with the Declaration of Helsinki.

Participants were assigned either to a therapy group or a waiting‐list control group. The participants in the therapy group underwent psychotherapy between the first and second assessment; participants in the waiting‐list control group did not undergo therapy, but had a similar time interval between the two assessments. All participants in the therapy group completed therapy. However, not all participants completed both assessments, and neuroimaging data from the second assessment were available for six individuals in the therapy group and nine individuals in the control group.

### 2.2 Questionnaires

To assess duration of military deployment and experiences during deployment, participants completed the Mental Health Advisory Team's Combat Experiences Scale (MHAT‐CES; Hoge et al., 2004; Wittchen, Gloster, Beesdo, Schönfeld, & Perkonigg, 2009), a 33‐item questionnaire assessing the type and frequency of combat‐related events during military deployment, as well as a study‐specific questionnaire that included items on the number and duration of military deployments. MHAT‐CES data were available for ten individuals in the therapy group and six individuals in the control group.

To assess psychological symptoms, prior to each neuroimaging session, participants also completed German versions of the following self‐report questionnaires; the Brief Symptom Inventory, the Posttraumatic Diagnostic Scale, the Interpretation of PTSD Symptoms Inventory, and the Posttraumatic Cognitions Inventory.

The Brief Symptom Inventory (BSI; Derogatis & Melisaratos, 1983) is a 53‐item self‐report questionnaire measuring nine symptom dimensions of psychological distress (e.g., somatization, depression, anxiety, and hostility). Each item was rated on a five‐point scale ranging from 0 (not at all) to 4 (extremely), based on the intensity of distress over the past week. For the BSI, we utilize the Global Severity Index (GSI) score, as this is recommended as the single best indicator of current psychological distress levels and is highly reliability over time, with a test–retest coefficient of 0.90 (Derogatis & Melisaratos, 1983). The GSI is calculated by taking the sum of the 53 items.

The Posttraumatic Diagnostic Scale (PDS; Foa, Cashman, Jaycox, & Perry, 1997) is designed to aid in the diagnosis of PTSD and assess symptom severity. As part of the PDS, respondents rated 17 items representing the main symptoms of PTSD experienced in the past 30 days, on a four‐point scale. The PDS has both strong internal consistency (Cronbach's α = 0.92) and good 3‐week test–retest reliability (κ = 0.74; Foa et al., 1997).

The Interpretation of PTSD Symptoms Inventory (IPSI; Clohessy & Ehlers, 1999) assesses appraisal of PTSD symptomatology over the past month, rated from 1 (totally disagree) to 7 (totally agree) and has good internal consistency (Cronbach's α = 0.93; Halligan, Michael, Clark, & Ehlers, 2003).

The Posttraumatic Cognitions Inventory (PTCI; Foa, Ehlers, Clark, Tolin, & Orsillo, 1999) is a 33‐item questionnaire on negative post‐trauma appraisals over the past month, relating to the self, the world, and self‐blame, rated from 1 (totally disagree) to 7 (totally agree). The PTCI has both strong internal consistency (Cronbach's α = 0.97) and high 3‐week test–retest reliability (Spearman's ρ correlation = .85).

Due to missing data, at the second assessment, BSI and PDS data were available for nine individuals in the therapy group and seven individuals in the control group, and IPSI and PTCI data were available for nine individuals in the therapy group and six individuals in the control group.

### 2.3 Psychotherapy

Trauma‐specific psychotherapy was performed by psychotherapists and psychotherapy‐specialized senior psychiatrists in single sessions using Eye Movement Desensitization and Reprocessing Therapy (EMDR). EMDR is one of the most common therapeutic interventions for PTSD (Foa, Keane, & Friedman, 2000) and is known to effectively reduce symptoms in the majority of individuals directly following completion of therapy (Seidler & Wagner, 2006; Shapiro, 1989, 1996). Participants completed two individual trauma‐specific EMDR sessions of 120 min each per week. During each EMDR session, the participant is asked to imagine the traumatic event, along with all accompanying emotional sensations while simultaneously engaging in periodic eye movements by attending to a moving visual stimulus controlled by the therapist. Furthermore, maladaptive, destructive cognition is identified, edited and corrected, and replaced by constructive cognition. The therapeutic goal is to integrate the traumatic event as a past event into the patient's life history, and in many cases, post‐traumatic growth, or positive change experienced as a result of a struggle with adversity, is also achieved. In addition to trauma‐focused individual therapy detailed above, participants also completed psychoeducation group sessions (250 min per week), occupational therapy (100 min per day), relaxation therapy (progressive muscle relaxation, Jacobson, 1938, 50 min per day), sports therapy (50 min per day), and physiotherapy (100 min per week).

### 2.4 Scanning procedure

Structural images were collected with a 3 Tesla Siemens Magnetom TrioTim scanner (Erlangen, Germany) using a standard 12‐channel head coil. The images were obtained using a three‐dimensional T1‐weighted magnetization prepared gradient‐echo sequence (MPRAGE) based on the ADNI protocol (http://www.adni-info.org; repetition time = 2,500 ms; echo time = 4.77 ms; TI = 1,100 ms, acquisition matrix = 256 × 256 × 176, flip angle = 7; 1 × 1 × 1 mm^3^ voxel size).

### 2.5 MRI Data analysis

Structural data were processed with voxel‐based morphometry (VBM8, http://dbm.neuro.uni-jena.de/vbm.html) and statistical parametric mapping (SPM8, http://www.fil.ion.ucl.ac.uk/spm) using default parameters running on MATLAB 9.1 (MathWorks, Sherborn, MA, USA). VBM is a neuroimaging analytic technique that allows investigation of focal differences in brain anatomy based on statistical parameter mapping of structural images. It involves bias correction, tissue classification, and affine registration. Images were normalized to the Montreal Neurological Institute (MNI) space using the ICBM152 template (Mazziotta et al., 2001) and segmented into gray matter, white matter, and cerebrospinal fluid based on voxel signal intensity and a priori expectation of tissue type based on anatomical location, using default parameters. Modulation was applied in order to preserve the volume of a particular tissue within a voxel by multiplying voxel values in the segmented images by the Jacobian determinants derived from the spatial normalization step. Images were smoothed with a full width at half maximum (FWHM) kernel of 8 mm.

**Figure 1:**
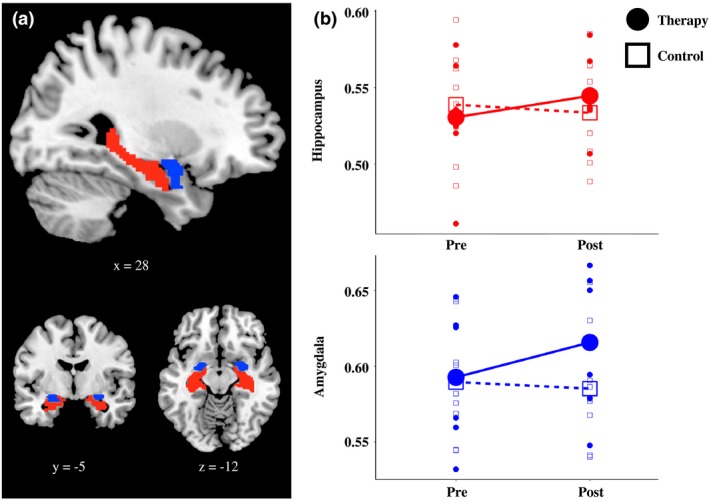
Bilateral hippocampal and amygdala gray matter volume. (a) Separate anatomical masks for the bilateral hippocampus (red) and amygdala (blue) were defined using the anatomical automatic labeling (AAL; Tzourio‐Mazoyer et al., 2002) template. Montreal Neurological Institute coordinates are provided below each brain image. (b) Gray matter values were extracted for the bilateral anatomical hippocampus and amygdala, and differences between the therapy and control group were assessed using a repeated measures ANOVA. The group by time interaction was significant for the hippocampus (*F*(1,13) = 7.33, *p *<* *.05, η_p_^2^ = 0.36) and close to significance for the amygdala (*F*(1,13) = 2.92, *p *=* *.11, η_p_^2^ = 0.18)

Region‐of‐interest analysis was conducted. To test the association between PTSD symptoms and brain regions implicated in PTSD, we extracted gray matter volume from the hippocampus and amygdala. Separate anatomical masks for the bilateral hippocampus and amygdala were defined using the anatomical automatic labeling (AAL; Tzourio‐Mazoyer et al., 2002) template (Figure 1a). The region of interest extraction (REX) toolbox (Whitfield‐Gabrieli, 2009) was used to extract gray matter volumes from the identified clusters.

## 3 RESULTS

Independent sample *t* tests were conducted to assess baseline differences in demographic variables between the therapy and control groups. The groups showed no significant differences in age, combat experiences, duration of military deployment, or in the time interval between pretherapy and post‐therapy assessments (Table 1).

**Table 1:** Demographic information collected at the pretherapy assessment

Variable	Therapy group (*n* = 11)	Control group (*n* = 9)	*t* test
Age (years)	27.4 ± 2.5	29.6 ± 8.8	*t*(18) = −0.79, *p *=* *.488
Military deployment (days)	211 ± 78.2	216 ± 117	*t*(18) = −0.10, *p *=* *.918
MHAT‐CES^a^	67.8 ± 31.5	54.3 ± 31.0	*t*(14) = 0.83, *p *=* *.419
Assessment interval (days)^b^	39.4 ± 19.9	49.7 ± 21.9	*t*(16) = −1.04, *p *=* *.316

Means and standard deviations are displayed for demographic information collected at the first assessment, along with independent samples *t* tests (two‐tailed).

a MHAT‐CES data were available for ten individuals in the therapy group and six individuals in the control group.

b Assessment interval data were available for nine individuals in the therapy group and nine individuals in the control group who completed either the second MRI or questionnaire assessment, or both. MHAT‐CES, Mental Health Advisory Team Combat Experiences Score.

Five participants did not undergo the second MRI assessment. Independent sample *t* tests were also conducted to assess baseline differences in demographic variables and psychological symptoms between dropouts and completers. The groups showed no significant differences in age, combat experiences, duration of military deployment, or scores on any of the psychological questionnaires.

Repeated measures ANOVA were conducted to assess change in psychological symptoms and gray matter volume between groups from pretherapy assessment to post‐therapy assessment. There was no main effect of time on gray matter in either the hippocampus or amygdala. However, there was a significant group by time interaction for the hippocampus (*F*(1,13) = 7.33, *p *<* *.05, η_p_^2^ = 0.36) and a nonsignificant trend for the amygdala (*F*(1,13) = 2.92, *p *=* *.111, η_p_^2^ = 0.18; Table 2 and Figure 1b). We also conducted paired *t* tests for each group separately. The increase in hippocampal volume from assessment 1 to assessment 2 in the therapy group was significant (*t*(5) = −2.11, *p *=* *.045, one‐tailed), while the decrease in the control group was not (*t*(8) = 1.39, *p *=* *.102, one‐tailed), while the increase in amygdala volume from assessment 1 to assessment to was not significant for either the therapy (*t*(5) = −1.316, *p *=* *.123, one‐tailed) or in the control group (*t*(8) = 0.690, *p *=* *.255, one‐tailed).

**Table 2:** Psychological questionnaires and region of interest gray matter

	Group (*n*)	Pre	Post	*t* test	RM ANOVA	
					Time	Time × group
BSI‐GSI	Therapy (9)	85.6 ± 26.4	70.2 ± 30.3	*t*(8) = 2.362, *p *=* *.023	*F*(1,14) = 4.69, *p *<* *.05, η_p_^2^ = 0.25	*F*(1,14) = 2.67, *p *=* *.13, η_p_^2^ = 0.16
	Control (7)	71.6 ± 38.3	69.4 ± 41.8	*t*(6) = 0.587, *p* = .289		
PDS	Therapy (9)	34.4 ± 7.28	28.1 ± 12.0	*t*(8) = 2.205, *p *=* *.029	*F*(1,14) = 10.01, *p *<* *.01, η_p_^2^ = 0.42	*F*(1,14) = 0.049, *p *=* *.82, η_p_^2^ = 0.003
	Control (7)	31.2 ± 14.1	24.0 ± 14.5	*t*(6) = 2.284, *p *=* *.031		
PTCI	Therapy (9)	132.8 ± 30.2	108.8 ± 33.1	*t*(8) = 2.767, *p *=* *.012	*F*(1,13) = 7.85, *p *<* *.05, η_p_^2^ = 0.38	*F*(1,13) = 0.05, *p *=* *.82, η_p_^2^ = 0.004
	Control (6)	125.5 ± 22.6	105.2 ± 46.9	*t*(5) = 1.402, *p *=* *.110		
IPSI	Therapy (9)	3.8 ± 1.3	3.2 ± 1.1	*t*(8) = 1.940, *p *=* *.044	*F*(1,13) = 2.97, *p *=* *.11, η_p_^2^ = 0.19	*F*(1,13) = 0.49, *p *=* *.50, η_p_^2^ = 0.036
	Control (6)	3.2 ± 1.0	3.0 ± 1.5	*t*(5) = 0.648, *p *=* *.273		
Hippocampus	Therapy (6)	0.53 ± 0.04	0.55 ± 0.03	*t*(5) = −2.11, *p *=* *.045	*F*(1,13) = 1.49, *p *=* *.24, η_p_^2^ = 0.10	*F*(1,13) = 7.33, *p *<* *.05, η_p_^2^ = 0.36
	Control (9)	0.54 ± 0.03	0.53 ± 0.03	*t*(8) = 1.39, *p *=* *.102		
Amygdala	Therapy (6)	0.59 ± 0.05	0.61 ± 0.05	*t*(5) = −1.316, *p* = .123	*F*(1,13) = 1.37, *p *=* *.26, η_p_^2^ = 0.095	*F*(1,13) = 2.92, *p *=* *.11, η_p_^2^ = 0.18
	Control (9)	0.59 ± 0.04	0.58 ± 0.04	*t*(8) = 0.690, *p *=* *.255		

Means and standard deviations are displayed for psychological questionnaire data and region‐of‐interest gray matter, collected at the first (Pre) and second (Post) assessment, separately for the therapy and waiting‐list control groups, along with paired samples *t* tests (one‐tailed) and repeated measures ANOVA. RM ANOVA, repeated measures analysis of variance; BSI‐GSI, Brief Symptom Inventory Global Severity Index; PDS, Posttraumatic Diagnostic Scale; PTCI, Posttraumatic Cognitions Inventory; IPSI, Interpretation of PTSD Symptoms Inventory; η_p_^2^, partial eta‐squared.

**Figure 2:**
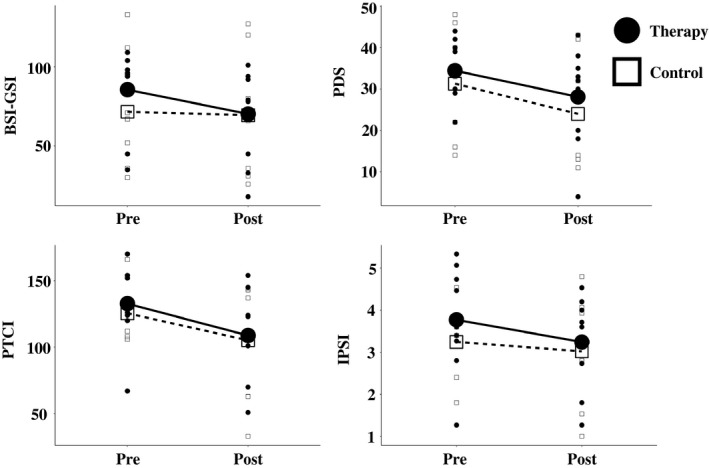
Psychological questionnaire scores. Psychological distress and PTSD symptoms were measured at both time points. Differences between the therapy and control group were assessed using a repeated measures ANOVA. There was a significant main effect of time for the BSI‐GSI (*F*(1,14) = 4.69, *p *<* *.05, η_p_^2^ = 0.25), PDS (*F*(1,14) = 10.0, *p *<* *.01, η_p_^2^ = 0.42), PTCI (*F*(1,13) = 7.85, *p *<* *.05, η_p_^2^ = 0.38) and a trend toward significant for the IPSI (*F*(1,13) = 2.97, *p *=* *.11, η_p_^2^ = 0.19). There was no significant group by time interaction for any of the questionnaire scores. BSI‐GSI, Brief Symptom Inventory Global Severity Index; PDS, Posttraumatic Diagnostic Scale; PTCI, Posttraumatic Cognitions Inventory; IPSI, Interpretation of PTSD Symptoms Inventory

Scores on all psychological questionnaire scores showed a decrease (either significant at *p *<* *.05 or a trend toward significance) between the pretherapy and post‐therapy assessments in both groups, but no group by time interaction was observed for any of the symptom scores (Table 2 and Figure 2). We also conducted paired samples *t* tests for each group separately. The therapy groups showed a significant reduction across all questionnaires (BSI; *t*(8) = 2.362, *p *=* *.023, one tailed, PDS; *t*(8) = 2.205, *p *=* *.029, one tailed, IPSI; *t*(8) = 1.940, *p *=* *.044, PTCI; *t*(8) = 2.767, *p *=* *.012), while the control group only showed a significant reduction on the PDS questionnaire (BSI; *t*(6) = 0.587, *p *=* *.289, one tailed, PDS; *t*(6) = 2.284, *p *=* *.031, one tailed, IPSI; *t*(5) = 0.648, *p *=* *.273, PTCI; *t*(5) = 1.402, *p *=* *.110).

Analysis revealed no significant correlations between hippocampal or amygdala gray matter volume and psychological symptoms, either across all participants or separately for each group.

## 4 DISCUSSION

Patients with combat‐related PTSD were assessed with psychological questionnaires and structural MRI neuroimaging at two time points approximately 6 weeks apart. Individuals who underwent psychotherapy between the two assessments were compared with a waiting‐list control group. Psychological symptoms significantly decreased for both groups over time, but no group by time interaction was observed. At the neural level, gray matter volume was assessed in the hippocampus and amygdala, two regions previously implicated in PTSD. No significant effect of time on gray matter volume was found; however, a group by time interaction was observed; the therapy group showed an increase in hippocampal gray matter volume, while the control group did not.

The hippocampus is one of the only sites of neurogenesis in the adult human brain (Eriksson et al., 1998) and has been shown to be highly plastic to environmental effects (Cotman & Berchtold, 2002; Draganski et al., 2006; Kempermann, Kuhn, & Gage, 1997). Neurogenesis in the hippocampus has been shown to modulate forgetting (Akers et al., 2014), while therapeutic interventions for depression and anxiety disorders increase hippocampal neurogenesis (Malberg, Eisch, Nestler, & Duman, 2000; Perera et al., 2007), and hippocampal neurogenesis is required for the behavioral effects of antidepressants (Santarelli, 2003). As such, the hippocampus and hippocampal neuroplasticity may also play a key role in resilience and recovery from stress. This is supported by the current finding that hippocampal volume increased following psychological therapy.

The current findings suggest that in the short term, some PTSD symptoms may reduce, even without therapeutic intervention. Psychological symptoms reduced across all questionnaires for the therapy group, while scores on the PDS also reduced in the control group. Although the mechanisms underlying the reduction in symptoms in the waiting‐list control group remain unclear, one possibility is that some individuals experienced spontaneous remission. Reductions of PTSD symptoms have previously been observed in individuals who have not received treatment (Güveli et al., 2006; Kessler, 1995). In addition, reductions in PTSD symptoms may be longer lasting when accompanied with increases in hippocampal volume. Further longitudinal work with multiple assessment points after completion of therapy would be useful to assess how increases in hippocampal gray matter volume may relate to long‐lasting reductions of psychological symptoms.

In a study with a similar design by Lindauer et al. (2005), patients in a therapy group showed a significant reduction in symptoms, but no change in hippocampal or amygdala volume compared with a waiting‐list control group. Although the approach and the sample size (*N* = 18) of the study by Lindauer and colleagues are comparable to the current study, one potential reason for the discrepancy between the results may be due to the use of different populations. In the current study, all participants were male, had experienced combat‐related trauma, and the mean age was 28 (*SD* = 6), while in the study by Lindauer and colleagues, over 50% of the participants were female, participants reported exposure to interpersonal violence or accidents or disasters, and the average age was 40 (*SD* = 9). Age and gender are known to play a key role in hippocampal neuroplasticity (Kuhn, Dickinson‐Anson, & Gage, 1996; Lisofsky et al., 2015) and resilience to stress (McEwen, 2002; McEwen & Morrison, 2013), while military and civilian populations have been shown to differ in risk factors (Brewin, Andrews, & Valentine, 2000) and response to psychotherapy (Bradley, Greene, Russ, Dutra, & Westen, 2005). If the effects of psychological therapy on the hippocampus are mediated by factors such as age, gender, or type of trauma, then the use of more homogeneous samples may be preferable to delineate these effects.

One should also note that the magnetic field strength employed by the two studies differs. Lindauer et al. (2005) analyzed images from a 1.5 Tesla scanner, while in the current study, images were collected using a 3 Tesla scanner. As such, the higher signal‐to‐noise ratio of the images in the current study, as well as other advancements in MRI image acquisition over the last decade, may have provided us with greater sensitivity to detect small effect sizes.

Participants were assessed twice, prior to therapy and directly following therapy, with an interval of approximately 6 weeks between assessments. Recent work on human brain plasticity with multiple neuroimaging assessments has demonstrated that brain regions undergo a period of expansion followed by renormalization during training interventions (Wenger, Brozzoli, Lindenberger, & Lövdén, 2017; Wenger et al., 2016), and that after a period of 6 weeks, gray matter volume has already begun to renormalize following initial expansion (Wenger et al., 2016). Although this work has focused on skill acquisition, it is also possible that therapeutic interventions may have stronger or wider effects on the brain at earlier stages in the treatment. Future studies may seek to incorporate multiple assessments during the therapy period to allow closer tracking of therapy‐related gains, and this may reveal greater effects than those currently observed.

The study has a number of potential limitations. Not all participants were available for follow‐up assessment. Although completers and dropouts did not differ significantly in their demographic or psychological characteristics at the initial assessment, there may be some nonrandom selection effects that are not captured by these variables. One should note that the size of the participant group may also limit the generalizability of the current findings. Given the relatively small sample size and explorative nature of this pilot study, we have interpreted both results significant at *p *<* *.05, and results that show a trend toward significance, as these findings may prove informative for future studies. In addition, due to the current sample size, we only conducted a region‐of‐interest analysis, which necessarily restricts analysis toward a priori hypothesized regions. Future work may seek to replicate the current findings with larger samples and extend them using a whole‐brain approach.

To our knowledge, this is the first study to compare combat‐related PTSD patients before and after therapy to a waiting‐list control group. In contrast to previous work, we find evidence for increases in gray matter volume in the hippocampus in response to psychological therapy. In the current study, we assessed combat‐exposed young adult males, while previous work assessed both male and female civilians with a larger age range. It is possible that age, gender, and military status may mediate the effect of psychological therapy on gray matter volume. Future work may seek to define and compare neural changes following therapy in female, older adult, and civilian populations.

## CONFLICT OF INTEREST

None declared.
